# Polyelectrolyte‐Enrobed Cancer Cells in View of Personalized Immune‐Therapy

**DOI:** 10.1002/advs.201700050

**Published:** 2017-05-02

**Authors:** Lien Lybaert, Keun Ah Ryu, Riet De Rycke, Alfred C. Chon, Olivier De Wever, Karim Y. Vermaelen, Aaron Esser‐Kahn, Bruno G. De Geest

**Affiliations:** ^1^ Department of PharmaceuticsGhent University9000GhentBelgium; ^2^ Department of ChemistryUniversity of California92618IrvineCAUSA; ^3^ VIB Inflammation Research Centerand Department of Biomedical Molecular BiologyGhent University9052GhentBelgium; ^4^ Department of Plant Systems BiologyVIB and Department of Plant Biotechnology and BioinformaticsGhent University9052GentBelgium; ^5^ Laboratory of Experimental Cancer ResearchGhent University9000GhentBelgium; ^6^ Tumor Immunology LaboratoryDepartment of Respiratory MedicineGhent University Hospital9000GhentBelgium

**Keywords:** dendritic cells, immunotherapy, microparticles, polyelectrolytes

## Abstract

Targeting the immune system with a personalized vaccine containing cues derived from the patient's malignancy might be a promising approach in the fight against cancer. It includes neo‐antigens as well as nonmutated tumor antigens, preferentially leading to an immune response that is directed to a broader range of epitopes compared to strategies involving a single antigen. Here, this paper reports on an elegant method to encapsulate whole cancer cells into polyelectrolyte particles. Porous and nonaggregated microparticles containing dead cancer cells are obtained by admixing mannitol and live cancer cells with oppositely charged polyelectrolytes, dextran sulfate (anionic polysaccharide), and poly‐l‐arginine (cationic polypeptide) prior to atomization into a hot air stream. It shows that the polyelectrolyte‐enrobed cancer cells, upon redispersion in phosphate buffered saline buffer, are stable and do not release cell proteins in the supernatant. In vitro experiments reveal that the particles are nontoxic and strongly increase uptake of cell lysate by dendritic cells. In vitro assessment of antigen presentation by dendritic cells reveal the potential of the polyelectrolyte‐enrobed cancer cells as promotors of antigen cross‐presentation. Finally, it is demonstrated that the immunogenicity can be enhanced by surface adsorption of a polymer‐substituted TLR7‐agonist.

## 1 Introduction

Antitumor therapy that involves dendritic cells (DCs) to evoke a tumor‐specific immune response is an attractive alternative to classic chemo‐ and irradiation therapy as it avoids the side‐effects associated to the latter therapies.^1^, ^2^, ^3^, ^4^, ^5^ Unfortunately, several hurdles remain between laboratory practice and successful clinical translation. One approach—termed personalized antitumor immune‐therapy—involves the formulation of patient's own tumor‐derived components into an anticancer vaccine.^6^, ^7^

Personalized immune‐therapy implementing patient's own tumor tissue of the patient, collected from a biopsy or from surgery, might hold promise to raise the potency and tumor‐specific immunity of cancer vaccines. The design of patient‐derived cancer cell vaccines can involve three different methods, i.e., identification of neo‐antigens, preparation of tumor cell lysate or the use of intact cancer cells. The first approach requires analysis of the genome of patient‐derived cells to identify proteins that are absent from the normal human genome and exclusively rise from tumor‐specific mutations.^8^, ^9^ This method however is complex, labor intensive, and costly. In contrast to this first approach, preparation of cancer cell lysate from the cancer tissue of the patient is less complex and includes neo‐antigens as well as nonmutated tumor antigens, preferentially leading to a broader immune response.^7^, ^10^ Third, incorporation of intact tumor cells can be an interesting approach as in this case all cell components such as cell membrane proteins are also involved and when translated to whole tumor tissue, also offer the possibility to coencapsulate stromal proteins. Vaccines comprising autologous cell material can be an alternative for exisiting vaccines based on allogenic tumor cell lines involving GVAX, a granulocyte‐macrophage colony‐stimulating factor(GM‐CSF) gene‐transfected tumor cell vaccine, encountering human leucocyte antigen (HLA) mismatch resulting in an anti‐HLA reponse rather than a tumor antigen‐directed reponse.^11^

**Figure 1:**
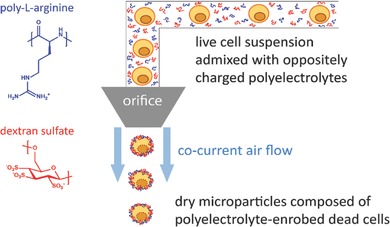
Schematic illustration of the production of polyelectrolyte‐enrobed whole cell microparticles. Live cells are mixed in aqueous solution with dextran sulfate (negatively charged polysaccharide) and poly‐l‐arginine (positively charged polypeptide). Atomization of this suspension in a heated air flow produces dry microparticles composed of single dead cells enrobed with a polyelectrolyte matrix.

In this paper, we describe a simple, yet efficient, strategy to formulate whole cancer cell lysate into microparticles and demonstrate that this process enhances antigen cross‐presentation by DCs. By admixing live cells in aqueous medium with oppositely charged polypeptides and polysaccharides followed by atomization into a hot air stream, a complex coacervate is formed surrounding the cells through spray drying. Evaporation of the water phase during the atomization process yields a dry powder composed of polyelectrolyte‐enrobed cancer cells. This approach is schematically illustrated in **Figure** **1**. Our method generates a whole cell‐based lysate within a single polyelectrolyte complex coacervate microparticle, and ensures—owing to the atomization/drying step—all cells to be dead in the final formulation. The latter avoids, upon administration, regrowth of new tumors due to residual living cells, as often the case with whole cell based lysates. In addition, our spray drying approach yields a dry particle formulation which can easily be stored over prolonged times and is highly attractive if one envisions multiple administrations over longer periods of time.^12^, ^13^, ^14^

## 2 Results and Discussion

As a model cancer cell line we used the murine Lewis lung carcinoma cell line (LLC.OVA) that is stably transfected with a nonsecreted, truncated form of ovalbumin (tOVA). The latter will act as tumor‐associated model antigen and allows a straightforward read‐out of the immunological response by OVA‐based assays.

### 2.1 Preparation of Polyelectrolyte‐Enrobed Cancer Cells

**Figure 2:**
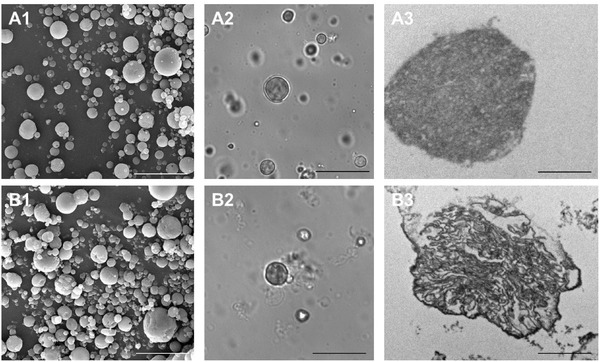
Scanning electron (1), optical (2), and (3) transmission electron microscopy images of A) empty microparticles and B) LLC.OVA containing microparticles. Scale bar is 20 µm in (1) and (2) and 2 µm in (3).

In a first series of experiments we examined whether spray drying of living cancer cells was feasible applying similar conditions as previously determined in our laboratories for spray‐drying of soluble proteins and polymers.^15^, ^16^, ^17^, ^18^ An LLC.OVA cell suspension, at a density of 60 × 10^6^ cells per 10 mL deionized water, was stirred on ice to minimize cell lysis and aggregation. Subsequently, mannitol, dextran sulfate (DEXS), and poly‐l‐arginine (P_L_ARG) were added a 40:4:5 (w/w) ratio. The role of mannitol is to enhance the microparticle recovery yield after the atomization step, to reduce protein denaturation and to generate porosity in the polyelectrolyte coacervate matrix which enhances protease influx and degradation of the matrix upon uptake by dendritic cells as shown in our earlier work.^16^ As control, microparticles were prepared without cells. Both formulations comprised a dry powder with an average recovery yield (calculated from the initial solid mixture amount) of ≈50%. Scanning electron microscopy (SEM) imaging (**Figure** **2** – panel 1) revealed that the cell‐containing particles exhibited a slightly more irregularly shape compared to the empty control particles. Redispersion of the microparticles in phosphate buffered saline (PBS; pH 7.4, 150 × 10^−3^ m NaCl) results both in case of cell‐containing microparticles and the empty control microparticles in the formation of a nonaggregated suspension, as confirmed by optical microscopy (Figure 2 – panel 2). This implies that the presence of cell material does not drastically change the spray drying process and stable cell‐containing microparticles can be obtained. It should be noted that the obtained microparticles are polydisperse which is related to the spray drying process and cannot be avoided. Transmission electron microscopy (TEM) imaging (Figure 2 – panel 3) revealed that the control particles had a relatively homogenous porous interior. By contrast, microparticles produced from LLC.OVA cells exhibit a more complex internal structure, which can likely be attributed to the presence of cellular components that yield high contrast on TEM, such as lipid‐rich domains, ER, and the nuclear envelope.

To assess whether each component in the formulation is required to obtain a stable microparticle suspension in PBS, we prepared control samples containing only cells, cells mixed with mannitol but no polyelectrolytes, and cells mixed with mannitol and only DEXS or P_L_ARG. As illustrated in Figure S1 (Supporting Information), none of these conditions were successful. Spray drying of cells only resulted in an extremely low yield, similar to what we previously observed for spray drying of proteins and polymers in absence of mannitol.^16^ Moreover, the recovered amount of material could not properly be redispersed again in PBS. Samples containing mannitol but no polyelectrolytes and samples containing mannitol and either one of both polyelectrolytes had decent yield (i.e., ≈50%), but only resulted in microparticles upon redispersion in PBS in case of cells/mannitol/P_L_ARG. However, the latter was highly aggregated, which we attribute to ionic gelation of the P_L_ARG by the divalent phosphate anions in PBS. When redispersion was performed in deionized water, again no microparticles were found. These findings clearly demonstrate the need for mannitol, DEXS and P_L_ARG to prepare microparticles at a sufficient recovery yield and with the ability to be properly redispersed in PBS.

**Figure 3:**
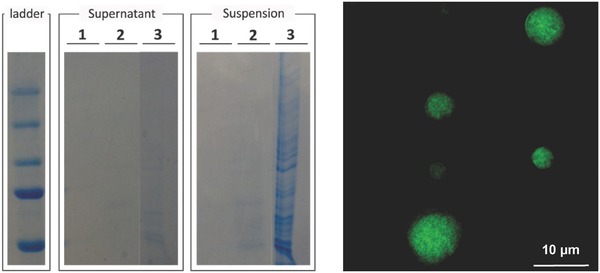
A) Assessment of the encapsulation efficiency upon redispersion in PBS via SDS‐PAGE recorded from the supernatant and suspension of (1) empty microparticles, (2) LLC.OVA containing microparticles, and (3) lyophilized LLC.OVA cells. B) Fluorescence microscopy image of a microparticle produced from the eGFP expressing CT5.3 cells.

Next, sodium dodecyl sulfate polyacrylamide gel electrophoresis (SDS‐PAGE) was used to analyze, whether upon redispersion in PBS, protein release from the microparticles occurred and thus to assess the encapsulation efficiency. We monitored the particle suspension itself and the supernatant comparing the cell‐containing microspheres with a lyophilized cell suspension obtained from the same amount of cells as used for the preparation of the cell‐containing microparticles. As shown in **Figure** **3**A (left panel – supernatant), relative to the lyophilized cell control sample (lane 3), no protein release was detected in the supernatant of the centrifuged microparticle suspension (lane 2). This clearly shows the strength of the formed polyelectrolyte matrix surrounding the cells as no cell proteins are released from the particles upon redispersion. In addition, we also visualized the microparticle suspension itself (Figure 3A: right panel – suspension) to rule out the possibility of the released proteins to be aggregated or precipitated. This revealed very little protein was present when the microparticle suspension itself was loaded onto the SDS‐PAGE gel (lane 2) compared to the lyophilized control sample (lane 3) and confirms the efficient encapsulation of the cell proteins. As a control, empty microparticles were also included (i.e., without cells) to exclude interference of the microparticle components. As shown in lane 1 of Figure 3A, the empty microparticles indeed could not be detected on the SDS‐PAGE gel. Additional UV–vis measurements were conducted to quantify the amount of released proteins in the supernatant upon redispersion of the cell‐containing microparticles. However, no proteins could be dectected in the supernatant confirming our previous findings obtained via SDS‐PAGE.

Further visual proof of successful encapsulation of cellular proteins into polyelectrolyte coacervate microparticles was gained by using the fluorescent eGFP expressing cell line CT5.3, a murine colon tumor‐derived cell line. A microparticle suspension prepared from this cell line exhibited a homogeneous green fluorescence throughout the microparticle volume (Figure 3B), without the presence of fluorescence in the surrounding medium. This was confirmed by fluorimetry and indicated an encapsulation efficiency of nearly 100%.

### 2.2 In Vitro Evaluation of Polyelectrolyte‐Enrobed Cancer Cells

**Figure 4:**
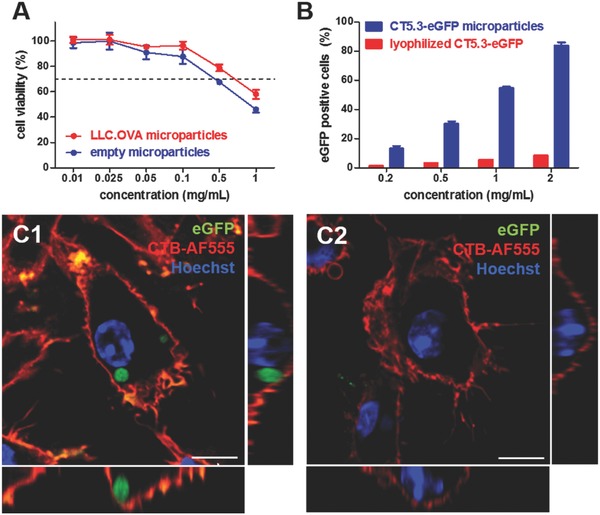
In vitro evaluation of spray dried cell‐derived polyelectrolyte microspheres on DC2.4 cells: A) MTT assay (n‐6). B) Flow cytometry analysis of uptake efficiency (n‐3). C) Confocal microscopy imaging of the interaction of the cell‐containing microspheres compared to lyophilized cells with DCs. The cell membrane is stained with AF555‐labeled cholera toxin B (CTB‐AF555) and the cell nuclei are stained with Hoechst. Scale bar represents 15 µm.

First, cytotoxicity of the microparticles was evaluated by MTT assay. This revealed the particles to be nontoxic up to a concentration of 0.5 mg mL^−1^ as depicted in **Figure** **4**A. Next, we assessed the in vitro uptake of the microparticles by the murine dendritic cell line DC2.4. For this purpose, microparticles produced from eGFP‐positive CT5.3 cells were used to allow for straightforward detection by fluorescence‐based methods. Flow cytometry was used to compare microparticle formulated cells with lyophilized cells. Note that both samples contained the same concentration of cell‐based material. From these data (Figure 4B) it was clear that formulated microparticles resulted in a more efficient cellular association of cell lysate in a dose‐dependent manner. Subsequently, confocal microscopy (Figure 4C) on similarly treated DC2.4 cells verified that the microparticles were indeed internalized by the DCs and were not merely bound to the cell membrane.

**Figure 5:**
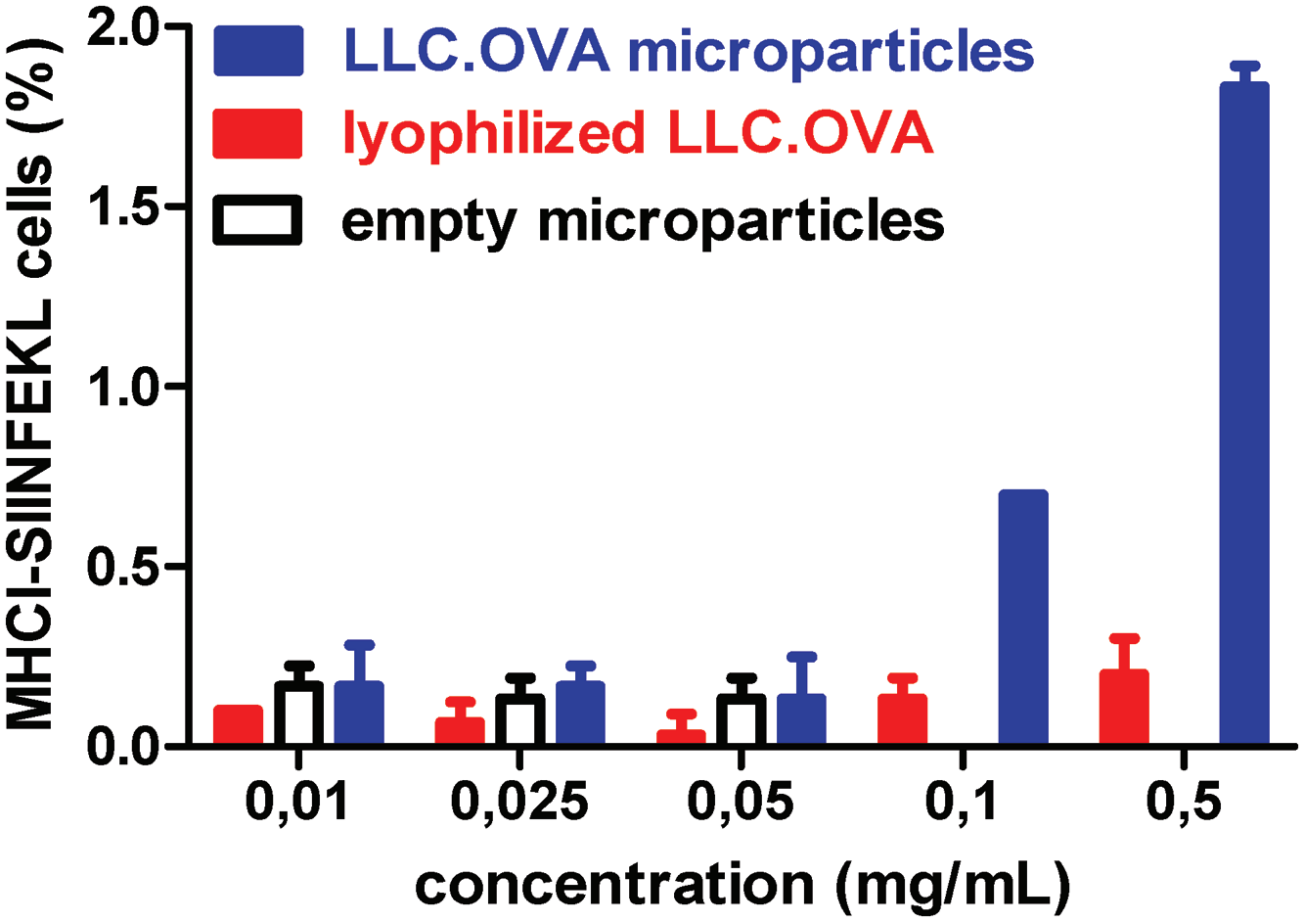
In vitro assessment of the MHC‐I cross‐presentation efficiency by DC2.4 cells.

Whereas soluble antigens are predominantly presented via MHC‐II peptide complexes by DCs to CD4+ T‐cells, formulation of soluble antigens into microparticles is known to enhance cross‐presentation via MHC‐I by DCs. The latter is essential for the priming of cytotoxic CD8+ T‐cells that hold the capacity to recognize tumor cells and eliminate these via secretion of perforin and granzymes.^17^, ^18^, ^19^ Here we investigated whether encapsulation of cell lysate into polyelectrolyte microparticles also promoted antigen presentation via MHC‐I. In this experiment, microparticles containing LLC.OVA cells were used. Successful processing and MHC‐I presentation of the ovalbumin, as model tumor‐associated antigen, in the cell lysate would enable a flow cytometric detection of the SIINFEKL OVA‐CD8+ epitope presented by MHC class I H‐2Kb molecules via antibody staining. After 48 h of incubation with different particle concentrations, DC2.4 cells were stained and analyzed by flow cytometry. **Figure** **5** clearly shows a dose‐dependent increase of the cross‐presentation efficiency when LLC.OVA cells were encapsulated in microparticles, whereas control experiments with lyophilized LLC.OVA cells did not show any significant cross‐presentation. Neither was this the case for empty microparticles.

### 2.3 Coformulation of Polyelectrolyte‐Enrobed Cancer Cells with Immune‐Stimulating Cues

After formulation of tumor antigens into particles to augment cross‐presentation, a potent cancer vaccine additionally requires coformulation of particulate antigens with immune‐stimulating cues to enhance the immunogenicity of the vaccine formulation.^20^, ^21^, ^22^, ^23^, ^24^ This is necessary because particle‐based formulation alone is insufficient for potent cytotoxic T‐cell (CTL)‐induction as this process requires three signals: [1] interaction between the T‐cell receptor and the MHC‐I presented antigen on the DC surface; [2] interaction between the CD28 T‐cell receptor and CD80 or CD86 on the DC surface; and [3] cytokine stimulation of T‐cells by DCs. The latter two signals can be mounted by triggering pathogen recognition receptors (PRRs) present at different location in DC, including cell surface and endosomal membranes and the cytoplasm. Amongst the multiple PRRs, Toll‐like receptors (TLRs) have been widely explored as target for molecular adjuvants to skew TH1‐driven immune responses and augment their amplitude and persistency. TLR7/8‐triggering is in particular attractive in the context of tumor vaccination as these receptors are present on the cell endosomal membrane in a wide range of both human and murine DC subsets. Triggering leads to elevated levels of type I IFN and IL‐12, which are key cytokines to promote TH1‐ and CTL‐responses required for potent antitumor immune responses.^25^, ^26^, ^27^ Interestingly, small molecule agonists of TLR7/8 based on guanosine analogues and imidazoquinolines have been identified and polymer‐conjugation of these molecules has recently been shown by us and others^28^, ^29^, ^30^ as an ideal strategy to reduce their systemic dissemination, thereby greatly enhancing their toxicity profile, and to enhance their adjuvanticity toward coadministered antigens.

**Figure 6:**
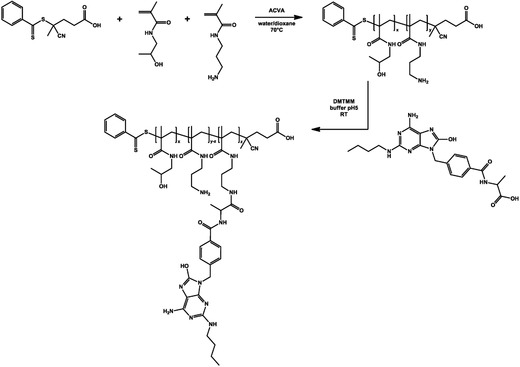
Synthesis of poly(HPMA‐APMA) and conjugation of the small molecule TLR7/8‐agonist CL264.

Here we used a polymer backbone composed of *N*‐(hydroxypropyl) methacrylamide (HPMA) and *N*‐(3‐aminopropyl) methacrylamide (APMA). Poly(HPMA‐APMA)) containing 80 HPMA and 20 APMA repeating units was synthesized via reversible addition‐fragmentation chain transfer (RAFT) polymerization as earlier reported.^31^ The primary amine moieties of this polymer were substituted with the TLR7/8‐agonist 2‐(4‐((6‐amino‐2‐(butylamino)‐8‐hydroxy‐9H_purin_9‐yl)methyl)benzamido)acetic acid yielding TLR7‐poly(HPMA‐APMA) (**Figure** **6**).

**Figure 7:**
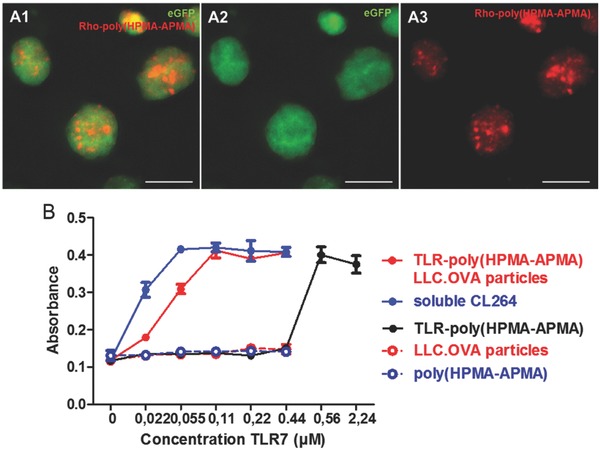
A) Confocal microscopy images of spray dried microspheres containing CT5.3‐eGFP cell material and rhodamine‐labeled poly(HPMA‐APMA). Scale bar represents 10 µm. B) RAW blue assay comparison of the soluble TLR7‐agonist, the polymer‐ligated TLR7‐agonist and the polyelectrolyte microspheres whether or not co‐formulated with TLR7‐poly(HPMA‐APMA).

The ability to coformulate this polymer with cell lysate into microparticles was tested using CT5.3‐eGFP cells and rhodamine labeled polymers that were synthesized by converting a small fraction of the poly(HPMA‐APMA) amino groups with rhodamine‐isothiocyanate. Confocal microscopy of the redispersed microparticles in PBS (**Figure** **7**A) clearly demonstrated the presence of the polymer, predominantly in a dotted pattern, likely due to complexation with the polyelectrolytes, whereas the eGFP signal was clearly visible throughout the whole microparticle volume. Subsequently, microparticles were produced containing TLR‐poly(HPMA‐APMA). A TLR‐reporter cell assay (i.e., RAW Blue) was performed to determine whether upon formulation into microparticles TLR‐triggering is still possible. Note that RAW Blue cells are engineered RAW 264.7 macrophages that express a broad range of PRRs and upon stimulation of these receptors produce secreted embryonic alkaline phosphatase, which can easily be detected by UV–vis spectrophotometry. As shown in Figure 7B, LLC.OVA containing particles as well as poly(HPMA‐APMA) did not evoke any activation evidenced by the lack of increase in absorbance relative to the negative PBS control. This suggested that the microparticles and the polymer on their own are poorly immunogenic.

Interestingly, the microparticles containing TLR‐poly(HPMA‐APMA) strongly promoted TLR‐activation almost equally efficiently as the soluble TLR7/8‐agonist opposed to the TLR‐containing polymer itself. This clearly shows the beneficial effect of particulate formulation of antigens together with TLR‐agonists as attractive vaccine carriers. The increase in potency can be attributed to more efficient uptake of the particulate vaccine formulation opposed to soluble vaccine which enables enhanced interaction of the TLR‐ligand with its receptor upon cell uptake. As the TLR‐poly(HPMA‐APMA) did not seem to induce any maturation up to a TLR7‐concentration of 0.44 × 10^−6^ m, higher concentrations were also tested in order to more clearly show the striking difference in activity of the TLR7‐ligand when formulated in particles or not. Indeed, the polymer alone only induces maturation starting from a TLR7‐concentration of 0.56 × 10^−6^ m whereas the TLR7‐containing particles already evoke activation at significantly lower concentrations. This decrease in efficiency of the polymer‐linked TLR7/8‐agonist can be attributed to steric hindrance of the polymer upon binding with the TLR‐receptor and/or partial shielding of the active site of the TLR7/8‐agonist. However, the latter is unlikely as both CL264 as well as other imidazoquinoline analogues have been conjugated at a similar position without fully abrogating their potency.^28^, ^29^, ^32^

## 3 Conclusion

In this paper we have reported on the formulation of whole cancer cells into solid polyelectrolyte‐based coacervate microparticles formed by the oppositely charged biologically inspired polyelectrolytes. Relative to cell lysate produced by lyophilization, microparticle‐formulated cells were internalized by DCs to a much larger extent. Using a cancer cell line that stably expresses OVA as model tumor‐associated antigen, we found that the antigen cross‐presentation efficiency by DCs was significantly enhanced in case of microencapsulated cells. We further demonstrated the ability to coencapsulate TLR7‐agonist‐ligated polymers into the microparticles and verified that TLR‐triggering can still occur. These findings pave the road for the development of whole cell based cancer vaccines that avoid the issue of tumor regrowth observed when using conditioned cancer cells and are more potent than soluble cell lysate based vaccines. In addition, since dry polyelectrolyte‐enrobed cancer cells were obtained via spray drying, this formulation is highly stable and does not require cold chain preservation prior to administration into the patient unlike current liquid vaccine formulations. Further experiments will involve in vivo assessment of the potency of the vaccine particles in triggering a robust antitumor immune response.

## 4 Experimental Section

*Materials*: Mannitol, dextran sulfate (10 kDa), poly‐l‐arginine hydrochloride (*M*_w_ > 70 kDa), 3‐(4,5‐dimethylthiazol‐2‐yl)‐2,5‐diphenyltetrazolium bromide (MTT) reagent, SDS, ethanol, dimethyl sulfoxide (DMSO), NaHCO_3_, and paraformaldehyde were obtained from Sigma‐Aldrich. Hydrochloric acid (HCl) 37% v/v and rhodamine‐NHS were purchased from Fischer Scientific. Dulbecco's modified Eagle medium (DMEM), RPMI 1640 medium, fetal bovine serum (EU qualified), penicillin/streptomycin (5000 U mL^−1^), sodium pyruvate (100 × 10^−3^ m), l‐glutamine (200 × 10^−3^ m), cell dissociation buffer (PBS based), PBS buffer (pH 7.2), Hoechst, cholera toxine B conjugates to AlexaFluor555 (CTB‐AF555), and Zeocin were obtained from Invitrogen. Laemli buffer (4x), 2‐Mercaptoethanol, Coomassie blue stain (G‐250), and 4–90 20% mini‐protean TGX gels were purchased from Bio‐rad whereas the pretreated Spectra/Por 7 dialysis membrane were purchased from Spectrumlabs. Quanti blue stain was obtained from Invivogen and antimouse OVA257‐264 (SIINFEKL) peptide bound to H‐2Kb PE antibody was purchased from eBioscience.

*Cell Lines: LLC: OVA Cell Line*: The LLC.OVA cell line was a kind gift from Prof. Karim Vermaelen (University of Ghent, Belgium). The cells were cultured in RPMI medium supplemented with 10% fetal bovine serum, 1% penicillin/streptomycin, 2 × 10^−3^ m l‐glutamine, and 1 × 10^−3^ m sodium pyruvate and incubated at 37 °C with 5% CO_2_ saturation.

*CT5.3‐eGFP Cell Line*: The CT5.3‐eGFP cell line^33^ was cultured in DMEM medium supplemented with 10% fetal bovine serum, 1% penicillin/streptomycin, 2 × 10^−3^ m l‐glutamine, and 1 × 10^−3^ m sodium pyruvate and incubated at 37 °C with 5% CO_2_ saturation.

*DC2.4 Cell Line*: The DC2.4 cell line was a kind gift from Dr. Kenneth Rock (University of Massachusetts, Boston, USA). The cells were cultured in RPMI medium supplemented with 10% fetal bovine serum, 1% penicillin/streptomycin, 2 × 10^−3^ m l‐glutamine, and 1 × 10^−3^ m sodium pyruvate and incubated at 37 °C with 5% CO_2_ saturation.

*RAW Blue Cell Line*: The RAW Blue cell line was purchased from Invivogen. The cells were cultured in DMEM medium supplemented with 10% heat‐inactivated fetal bovine serum, 1% penicillin/streptomycin, 2 × 10^−3^ m l‐glutamine, 1 × 10^−3^ m sodium pyruvate and 0.01% Zeocin and incubated at 37 °C with 5% CO_2_ saturation.

*Synthesis of Cancer Cell Polyelectrolyte Microspheres*: Prior to cell count, mannitol and DEXS were dissolved in 10 mL of lipopolysaccharide (LPS) free water to a concentration of 20 or 2 mg mL^−1^, respectively, and a 5 mg mL^−1^ solution of P_L_ARG was prepared in LPS free water. Next 60 × 10^6^ LLC.OVA cells were suspended in 15 mL of LPS free water and added to the mannitol–DEXS solution under stirring on ice. All handlings were performed in sterile conditions in a biohood to avoid endotoxin contamination. Subsequently, 5 mL of the poly‐l‐arginine solution was added dropwise under stirring to the cell suspension on ice prior to spray drying. Spray drying of the mixtures was performed on a lab‐scale Buchi B2902 spray‐dryer under sterile conditions. The latter involved presterilization of the spray dryer with ethanol and LPS free water prior to spray drying of the test samples. The spray‐dryer operated in cocurrent air flow at drying air temperature of 130 °C. After spray drying the yield was determined and the obtained powder was stored at ‐20 °C. The samples were visualized on a Leica DM2500P microscope equipped with a 40× (NA 0.75) objective, DIC filter, and a DFC360FX camera after reconstitution in water. The initial weight of the LLC.OVA cell suspension was determined after lyophilization to calculate the yield after spray drying and of the amount of lyophilized cells needed as control.

*Electron Microscopy: Scanning Electron Microscopy*: SEM was performed on a Quanta 200 FEG FEI instrument. Samples were deposited onto a silicon wafer and dried under a gentle nitrogen stream at ambient temperature. Prior to imaging, the samples were sputtered with a palladium/gold coating.

*Transmission Electron Microscopy*: TEM was performed on a JEOL 1010 instrument. Prior to imaging, samples were subjected to series of fixation (0.1 m Na cacodylate buffer (pH 7.2) containing 4% paraformaldehyde and 2.5% glutaraldehyde) and dehydration steps, embedded in epoxy resin and cut into ultrathin section using an ultramicrotome.

*Gel Electrophoresis (SDS‐PAGE)*: To analyze cell lysate encapsulation efficiency upon reconstitution of the particles after spray drying in LPS free water, gel electrophoresis was performed. The samples were diluted with a 1:9 2‐mercaptoethanol:Laemli sample buffer solution (4x), incubated for 5 min at 95 °C and loaded on 4%–20% precast gels. After the run (150 kV), visualization of the protein bands was achieved by incubation of the gels into Coomassie blue stain.

*MTT Assay*: Cell viability was assessed by the 3‐(4,5‐dimethylthiazol‐2‐yl)‐2,5‐diphenyltetrazolium bromide (MTT) assay. DC2.4 cells were seeded in 96‐well plates at a density of 5 × 10^4^ cells mL^−1^ (total volume 100 µL) in sixfold. Subsequently, the cells were incubated with different concentrations of microspheres and lyophilized LLC.OVA cells and cultured for 24 h followed by addition of 40 µL of the MTT reagent (1 mg mL^−1^). After an incubation period of 2–3 h the formed formazan crystals were dissolved in 100 µL of a 10% m/v SDS/0.01 m HCl solution overnight protected from light. The absorbance was measured by a microplate reader at 570 nm. As a negative and positive control PBS buffer and DMSO, respectively, were added to the wells.

*In Vitro Cell Uptake Assay*: DC2.4 cells were seeded in a 24‐well plate at a density of 0.15 × 10^6^ cells mL^−1^ one day before the cells were pulsed with fluorescent particles at different concentrations. After 24 h of incubation, the cells were dissociated using cell dissociation buffer followed by centrifugation for 5 min at 200 G at 0 °C. After resuspension, the samples were stored on ice and measured on a BD Accuri C6 flow cytometer. The data were analyzed using FlowJo.

*Confocal Microscopy*: DC2.4 cells were seeded at a density of 0.4 × 10^6^ cells mL^−1^ in a glass bottom Will‐co dish and incubated overnight. Next, the fluorescent particles were added, incubated for 24 h, and fixated in a 2% paraformaldehyde solution for 10–15 min. The cells were subsequently washed and simultaneously stained by CTB‐AF555 and Hoechst for 1 h at room temperature. Finally, the samples were washed with PBS and imaging on a confocal microscope (Leica DMI6000 B inverted 241 microscope) equipped with an oil immersion objective (Zeiss, 63×, 242 NA 1.40) and attached to an Andor DSD2 confocal scanner.

*In Vitro MHC‐I Presentation Assay*: DC2.4 cells were seeded at a density of 0.2 × 10^6^ cells mL^−1^ in a 24‐well culture plate and incubated overnight followed by incubation with different concentrations of the samples. After 48 h at 37 °C the positive control (SIINFEKL) was added in triplicate in a concentration of 1 µg mL^−1^, 1 h prior to staining with SIINFEKL‐MHC‐I PE‐labeled antibody for 30 min on ice protected from light. Subsequently, the samples were centrifuged for 5 min at 200 G at 4 °C, resuspended in PBS and analyzed with flow cytometry.

*Fluorescent Labeling of Poly(HPMA‐APMA)*: Poly(HPMA‐APMA) was incubated with an equimolar amount of rhodamine‐NHS in 0.1 m NaHCO_3_ buffer overnight under continuous stirring. Subsequently, the obtained mixture was dialyzed against deionized water for 3 d (MWCO 3.5 kDa) and lyophilized.

*Polymer Synthesis and Conjugation of CL264*: Poly(HPMA‐APMA), dissolved in LPS free water, was incubated with an equimolar amount of TLR7‐ligand CL264 to APMA units overnight under continuous stirring at room temperature in the presence of 1.5 m excess of DMTMM. After 24 h incubation the reaction mixture was dialyzed against LPS free water (MWCO 3.5 kDa) for 1 d and lyophilized.

*In Vitro RAW Blue Assay*: RAW Blue macrophages were seeded in a 96 well round bottom plate at a density of 0.5 × 10^6^ cells mL^−1^ and immediately pulsed with the desired concentrations of the test compounds in sixfold. As a negative control PBS was added. After 24 h incubation, 50 µL of the supernatant was transferred into a 96 well flat bottom plate and incubated with 150 µL of Quanti blue solution. After 3 to 6 h incubation at 37 °C the color change absorbance was measured with a plate reader at 620–655 nm.

## Conflict of Interest

The authors declare no conflict of interest.

## Supporting information

Supplementary
