# Supplementary material for: Polyelectrolyte‐Enrobed Cancer Cells in View of Personalized Immune‐Therapy
Source: Adv Sci (Weinh). 2017 May 2;4(6):1700050. doi: 10.1002/advs.201700050 (PMC5473321; doi:10.1002/advs.201700050)
Supplement: Supplementary file 1 — Supplementary [file ADVS-4-na-s001.pdf]

Copyright WILEY-VCH Verlag GmbH & Co. KGaA, 69469 Weinheim, Germany, 2017.

Supporting Information

for *Adv. Sci.,* DOI: 10.1002/advs.201700050

Polyelectrolyte-Enrobed Cancer Cells in View of Personalized Immune-Therapy

Lien Lybaert, Keun Ah Ryu, Riet De Rycke, Alfred C. Chon, Olivier De Wever, Karim Y. Vermaelen, Aaron Esser-Kahn, and Bruno G. De Geest**

SUPPORTING INFORMATION

Polyelectrolyte-enrobed cancer cells in view of personalized immune-therapy.

L . Lybaert[1], Keun Ah Ryu[2], Riet De Rycke[3,4], Alfred Chon[2], Olivier De Wever[5], Karim Y. Vermaelen[6], Aaron Esser-Kahn[2*] and Bruno G. De Geest[1*]

1 Department of Pharmaceutics, Ghent University, Ghent, Belgium

2 Department of Chemistry, University of California, Irvine (CA), USA

3 VIB Inflammation Research Center, Ghent University, Ghent, Belgium and Department of Biomedical Molecular Biology, Ghent University, 9052 Ghent, Belgium

4 Department of Plant Systems Biology, VIB, Ghent, Belgium and Department of Plant Biotechnology and Bioinformatics, Ghent University, 9052 Gent, Belgium

5 Laboratory of Experimental Cancer Research, Ghent University, Ghent, Belgium

6 Tumor Immunology Laboratory, Department of Respiratory Medicine, Ghent University Hospital, Ghent, Belgium

* Coressponding authors: aesserka@uci.edu, br.degeest@ugent.be

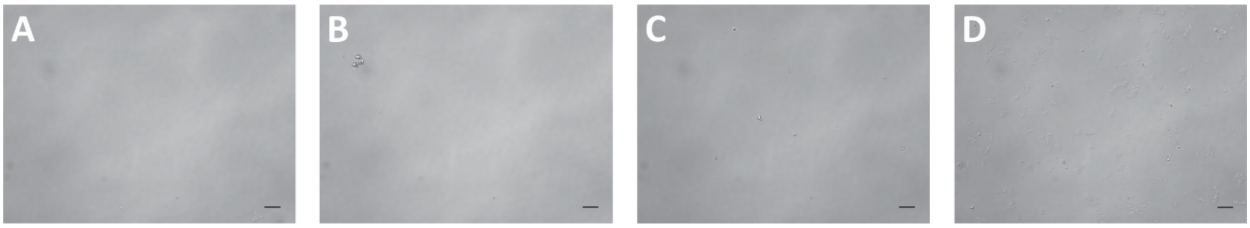

Figure S1. Microscopic imaging of spray dried control samples containing. (A) only cell material. (B) cell material and mannitol. (C) cell material, mannitol and dextran sulfate. (D) cell material, mannitol and poly-L-arginine. Scale bar is 10 µm.
